# Novel Intermolecular Iterative Mechanism for Biosynthesis of Mycoketide Catalyzed by a Bimodular Polyketide Synthase 

**DOI:** 10.1371/journal.pbio.0060163

**Published:** 2008-07-08

**Authors:** Tarun Chopra, Srijita Banerjee, Sarika Gupta, Gitanjali Yadav, Swadha Anand, Avadhesha Surolia, Rajendra P Roy, Debasisa Mohanty, Rajesh S Gokhale

**Affiliations:** National Institute of Immunology, New Delhi, India; University of California, San Diego, United States of America

## Abstract

In recent years, remarkable versatility of polyketide synthases (PKSs) has been recognized; both in terms of their structural and functional organization as well as their ability to produce compounds other than typical secondary metabolites. Multifunctional Type I PKSs catalyze the biosynthesis of polyketide products by either using the same active sites repetitively (iterative) or by using these catalytic domains only once (modular) during the entire biosynthetic process. The largest open reading frame in Mycobacterium tuberculosis, *pks12*, was recently proposed to be involved in the biosynthesis of mannosyl-β-1-phosphomycoketide (MPM). The PKS12 protein contains two complete sets of modules and has been suggested to synthesize mycoketide by five alternating condensations of methylmalonyl and malonyl units by using an iterative mode of catalysis. The bimodular iterative catalysis would require transfer of intermediate chains from acyl carrier protein domain of module 2 to ketosynthase domain of module 1. Such bimodular iterations during PKS biosynthesis have not been characterized and appear unlikely based on recent understanding of the three-dimensional organization of these proteins. Moreover, all known examples of iterative PKSs so far characterized involve unimodular iterations. Based on cell-free reconstitution of PKS12 enzymatic machinery, in this study, we provide the first evidence for a novel “modularly iterative” mechanism of biosynthesis. By combination of biochemical, computational, mutagenic, analytical ultracentrifugation and atomic force microscopy studies, we propose that PKS12 protein is organized as a large supramolecular assembly mediated through specific interactions between the C- and N-terminus linkers. PKS12 protein thus forms a modular assembly to perform repetitive condensations analogous to iterative proteins. This novel intermolecular iterative biosynthetic mechanism provides new perspective to our understanding of polyketide biosynthetic machinery and also suggests new ways to engineer polyketide metabolites. The characterization of novel molecular mechanisms involved in biosynthesis of mycobacterial virulent lipids has opened new avenues for drug discovery.

## Author Summary

Polyketide synthases (PKSs) form a large family of multifunctional proteins involved in the biosynthesis of diverse classes of natural products. Mycobacterium tuberculosis (Mtb) exploits these polyketide biosynthetic enzymes to synthesize complex lipids, many of which are essential for its virulence. PKSs utilize two common mechanistic themes to produce these metabolites: (1) modular—in which each set of catalytic sites is used only once during the entire biosynthetic process and (2) iterative—in which the same set of active sites is used repeatedly. Our study with PKS12 protein from Mtb (the largest protein in this genome) reveals a third mechanism for polyketide biosynthesis. In this hybrid “modularly iterative” mechanism, PKS12 protein forms a supramolecular assembly to perform repetitive cycles of iterations. The protein assembly is formed by specific intermolecular interactions between N- and C-terminus linkers, analogous to modular PKSs. Our study adds a new dimension to the existing catalytic and mechanistic versatility of PKSs, providing a new perspective on how metabolic diversity could be generated by different combinations of existing functional scaffolds.

## Abstract 3

A novel iterative biosynthetic mechanism for multifunctional polyketide synthases reveals how the metabolic diversity of this enzyme family can arise by using existing scaffolds in novel combinations.

## Introduction

Polyketide synthases (PKSs) are multifunctional enzymes that assemble simple CoA-thioesters to form complex metabolites. These proteins are classified as Type I, Type II, and Type III based on their protein architecture. Type I PKSs contain various catalytic domains on a single polypeptide chain, which are functionally grouped to form a module. These are further classified as modular and iterative. In modular type I PKSs, each set of catalytic domains is used once during the entire cycle of biosynthesis, whereas iterative PKSs repeatedly use these functional domains [1–5]. Type II PKSs, on the other hand, assemble different catalytic domains to form a noncovalent multienzyme complex [6,7]. Both these types of PKSs require a carrier domain on which intermediates are tethered as acyl-thioesters. Type III PKSs are quite different and are classified along with other PKSs due to their mechanistic similarity in the process of 2-carbon condensations [8,9]. Type I and II PKSs utilize three core catalytic domains, ketosynthase (KS), acyl transferase (AT), and acyl carrier protein (ACP), to carry out decarboxylative condensations. The β-keto intermediates are processed by auxiliary domains; ketoreductase (KR), dehydratase (DH), and enoyl reductase (ER), that dictate the overall chemistry of the metabolite.

Research in recent years has provided new themes that enhance the ability of PKSs to produce a wide array of metabolites that could mediate processes of significant functional importance. For example, AT-less Type I PKSs have been characterized, which utilize a discrete AT domain to load extender units in *trans* [10]. Fungal Type I iterative PKSs suggest new mechanistic basis of selective utilization of auxiliary reductase and methyl transferase domains [11–13]. The catalytic and mechanistic versatility of mycobacterial PKSs in generating complex virulent lipids have also been recently recognized [3,14]. A family of fatty-acyl AMP ligases has been demonstrated to facilitate biochemical crosstalk between fatty acid synthases (FASs) and PKSs [15]. An emerging family of type III PKSs involved in biosynthesis of novel resorcinolic lipids has also been characterized from Azotobacter vinelandii and Neurospora crassa [16,17]. Since metabolic diversity far exceeds our present understanding of the biosynthetic capability of this family of proteins, it is tempting to speculate that many new mechanistic and organizational features of PKSs remain to be decoded.

In this study, we report a novel mechanism of iterative catalysis for a bimodular multifunctional PKS12 (Rv2048c) protein from M. tuberculosis. This protein is involved in biosynthesis of a phosphoglycolipid called mannosyl-β-1-phosphomycoketide (MPM) [18]. The chemical structure of mycoketide lipid chain has been proposed to originate from repetitive condensations of methylmalonate and malonate units with medium-chain fatty acyl starters. Involvement of PKS12 protein in the biosynthesis of MPMs has been previously supported by genetic studies. By using a combination of biochemical, computational, mutagenic, analytical ultracentrifugation, and atomic force microscopy (AFM) studies, we propose that PKS12 protein is organized in a large supramolecular assembly mediated through specific interactions between the N- and C-terminus linkers (Nter and Cter, respectively). This assembly of PKS12 protein provides evidence for the intermolecular iterative mechanism for mycoketide biosynthesis. Our studies thus provide the first evidence for a novel “modularly iterative” mechanism for biosynthesis by PKSs.

## Results

### Correspondence between Mycoketide Structure and PKS12 Catalytic Domains

**Figure 1:**
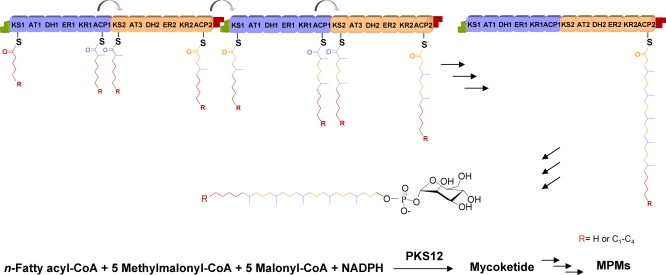
Involvement of PKS12 Protein in the Biosynthesis of Mannosyl-β-1-Phosphomycoketide A schematic representation of bimodular PKS12 protein from M. tuberculosis, which is required for the biosynthesis of mycoketide. The two modules of PKS12 are colored in blue (AT specificity for methylmalonyl CoA) and buff (AT specificity for malonyl CoA), and the N- and Cters are indicated in green and red, respectively. Individual domains are drawn as small boxes in the two modules. Mycoketide chain originates from repetitive condensations of methylmalonate and malonate units with medium-chain fatty acyl starters. This saturated chain is released from PKS12 by an unknown mechanism, which is followed by reduction, phosphorylation, and mannosylation to form MPM.

The lipid portion of MPM, also referred to as mycoketide, consists of saturated alkyl chain with methyl branching at every fourth carbon (Figure 1). The total number of carbons could vary from C_30_ to C_34_ in different mycobacterial species [18]. Due to its similarity with mannosyl-β-1-phosphodolichol (MPD), initial structural characterization had suggested an isoprenoid mode of biosynthesis [19]. Based on genetic studies, PKS12 protein was recently shown to be involved in the biosynthesis of mycoketide. It is proposed that the biosynthesis would involve an iterative mode of catalysis. In mycobacteria, PKS12 is the largest protein, comprising 12 catalytic domains constituting two modules (Figure 1). Both these modules contain a complete set of catalytic and auxiliary domains that could add 2-carbon ketide units and also simultaneously carry out a three-step reduction to a saturated alkyl chain. Since mycoketide chemical structure contains branching at every alternate ketide unit, a possible iterative mechanism would involve alternate use of two modules wherein one module would condense a branched C_3_ ketide unit and the next would add a C_2_ unit. This would indicate that the two modules of PKS12 would be specific for methylmalonate and malonate extender units, respectively. The AT domains of PKSs characteristically show strict substrate specificity; however, there are examples in which the same AT domain could utilize both malonate and methylmalonate units [20]. Such promiscuity of AT domains in PKS12 could result in a complex mechanism of biosynthesis. We therefore decided first to dissect the specificity dictated by the two AT domains of PKS12 protein.

### Heterologous Expression of 431-kDa PKS12 Protein and Characterization of AT Domain Specificities

**Figure 2:**
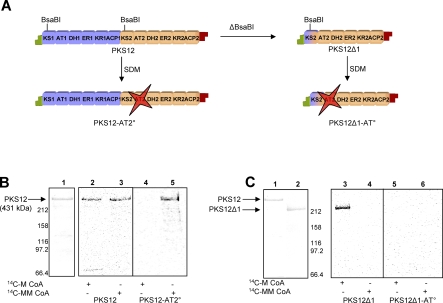
Determination of Specificities of the AT Domains and Construction of Single-Module PKS12Δ1 Protein (A) The PKS12Δ1 protein was constructed by deleting module 1 from the clone encoding PKS12 protein. The DNA encoding KS domains from module 1 and module 2 contains a conserved BsaBI restriction endonuclease site, which facilitated module 1 deletion. PKS12Δ1 retains N- and Cters of PKS12 protein. PKS12 AT2 null mutant (PKS12AT2°) and PKS12Δ1 AT null mutant (PKS12ΔAT°), both mutated at the catalytic Ser residue were constructed by using standard SDM protocols. The mutations in the domains are marked in red. (B) Labeling of PKS12 protein with radioactive extender units. Lane 1 shows the Coomassie-stained, purified PKS12 protein (431 kDa). The protein could be labeled with both ^14^C-MCoA (lane 2) and ^14^C-MMCoA (lane 3). AT2 (S2672A) point mutant of PKS12 is labeled by ^14^C-MMCoA (lane 5) and not by ^14^C-MCoA (lane 4) depicting specificity of module 1 for MMCoA and module 2 for MCoA. (C) Labeling of PKS12Δ1 protein with radioactive extender units. Lanes 1 and 2 show the Coomassie-stained, purified PKS12 (431 kDa) and PKS12Δ1 protein (234 kDa). PKS12Δ1 protein could be radiolabeled with ^14^C-MCoA (lane 3) but not with^14^C-MMCoA (lane 4). AT (S2672A) mutant protein of PKS12Δ1 does not incorporate any radioactive label (lanes 5 and 6).

The 12,456-bp *pks12* gene was cloned in T7 expression vector such that the protein would be expressed with a C-terminal His-tag. The N- and C- terminal ends were suitably engineered, and the central region of *pks12* was spliced from overlapping shotgun library of mycobacterial D8 BAC clone. The phosphopantetheinylation of the ACP domain of PKS12 was achieved by coexpression of pRSG56 (a surfactin phosphopantetheinyl transferase [Sfp] expression plasmid) in BL21-DE3 strain of Escherichia coli [21,22]. Purification by Ni^2+^-NTA affinity chromatography followed by anion exchange chromatography showed a protein band running above the 212-kDa marker on the SDS-PAGE (Figure 2B, lane1). Since E. coli protein biosynthetic machinery is not programmed to synthesize such large proteins, and it is not easy to estimate exact molecular weight from SDS-PAGE for such large proteins, we performed peptide mapping by using mass spectrometry. Our analysis showed good coverage over the entire length of the protein, confirming expression of complete PKS12 protein. The specificity of AT domains was probed by using ^14^C malonyl coenzyme A (MCoA) and ^14^C methylmalonyl coenzyme A (MMCoA). SDS-PAGE gel-binding assays clearly showed that PKS12 could be radiolabeled with both of these substrates (Figure 2B, lanes 2 and 3). However, the specificity of individual AT domains to load extender units could not be determined. We therefore decided to engineer a single-module construct of PKS12.

### Engineered Single-Module PKS12 Protein and Assignment of AT Domain Specificity

Based on previous understanding of engineering single-module PKS proteins, we utilized the in-frame BsaBI restriction sites present in the *pks12* gene to delete module 1. This engineered PKS12Δ1 protein retained both the Nter and Cter and all the catalytic domains of module 2 (Figure 2A). PKS12Δ1 protein was expressed and purified analogous to PKS12 (Figure 2C, lane 2). The specificity of AT domain was again investigated by using radiolabeled substrates. PKS12Δ1 protein specifically utilized MCoA and no labeling of protein could be identified with MMCoA (Figure 2C, lanes 3 and 4). The specificity of this labeling was further confirmed by making a point mutation of the catalytic Ser amino acid residue of the AT domain. The S2672A null mutant protein, PKS12Δ1-AT°, was now incapable of loading any of these two extender units (Figure 2C, lanes 5 and 6). These studies clearly demonstrate specificity of module 2 for malonate unit and suggest that the module 1 of PKS12 protein would add a methylmalonate unit.

In order to unambiguously determine the specificity of AT1, we constructed an AT2 null mutant of PKS12, PKS12-AT2° (Figure 2A). This mutant protein could be specifically radiolabeled with ^14^C MMCoA and not with ^14^C MCoA (Figure 2B, lanes 4 and 5) thus demonstrating specificity of module 1 for MMCoA. These studies clearly demonstrate that the biosynthesis of mycoketide would involve alternate use of module 1 and module 2, respectively. The final assembly would involve five such cycles of iterations, and in terms of thiotemplate-based enzymology, would require transfer of growing polyketide chain from the ACP domain of module 2 to the KS domain of module 1. Such bimodular iterations during PKS biosynthesis have not been demonstrated. We therefore decided to investigate whether PKS12 protein by itself would be competent to produce mycoketide chain.

### Cell-Free Reconstitution of PKS12 and PKS12Δ1 Protein

**Figure 3:**
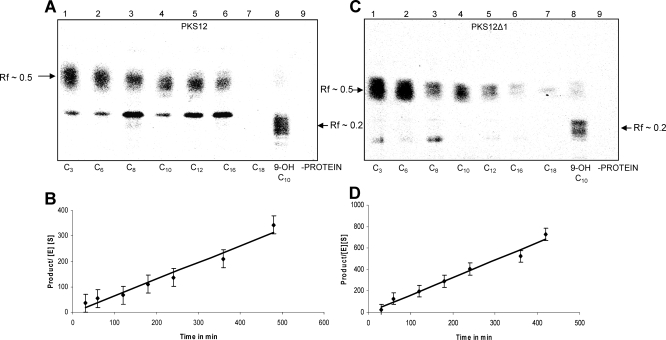
Biochemical Characterization of PKS12 and PKS12Δ1 Proteins (A)Radio-TLC characterization of PKS12 products. Lane 1 to 8 show the propionyl, hexanoyl, octanoyl, decanoyl, lauroyl, palmitoyl, stearoyl, and 9-hydroxy decanoyl N-acetyl cysteamine thioester primed products. No product is formed in the absence of protein (lane 9). (B) Time course of product formation catalyzed by PKS12 protein. The error bars correspond to three independent experiments, and the data points are an average of all three. (C) Radio-TLC characterization of PKS12Δ1 products. Lane 1 to 8 show the propionyl, hexanoyl, octanoyl, decanoyl, lauroyl, palmitoyl, stearoyl, and 9-hydroxy decanoyl N-acetyl cysteamine thioester primed products. No product is formed in the absence of protein (lane 9). (D) Time course of product formation catalyzed by PKS12Δ1 protein. The error bars correspond to three independent experiments, and the data points are an average of all three.

The catalytic activity of PKS12 protein was analyzed by incubating various starters (C_6_–C_18_) acyl-N-acetyl cysteamines (NACs) with radiolabeled extender units MCoA, MMCoA, and NADPH. Since PKS12 protein does not contain a chain-releasing domain, the enzyme-bound products were released by alkali hydrolysis. The extracted products were analyzed by performing radio-thin layer chromatography (TLC) experiments. Autoradiography analysis showed a diffused band at an *R*_f_ of about 0.5 (Figure3A). When either of the extender units was omitted from the assays, such broad bands were not observed (Figure S1). PKS12 protein showed preference for small-/medium-chain starter fatty acyl substrates. Radioactive bands observed at lower *R*_f_ were degradative products of acyl-CoAs and varied from assay to assay. Enzymatic assays of PKS12 protein with 9-hydroxy decanoyl NAC-thioester shifted *R*_f_ of the final product to about 0.2 (Figure3A, lane 8). Hydroxyl modification at ω-position of fatty acids dramatically alters the *R*_f_ value of products owing to the polar nature of starter unit and is an interesting approach to confirm enzymatic activity. Furthermore, incubation of PKS12 protein with KS inhibitor cerulenin showed no radioactive bands (Figure S2). We speculated that the diffused radioactive band could correspond to different intermediates formed during various stages of iterations. Our previous studies with another single module iterative enzyme, mycocerosic acid synthase, had shown presence of similar diffused bands [20]. The apparent catalytic turnover (*k*_cat_/*K*_M_) of the final product was estimated to be 42.4 μM^−1^ sec^−1^ (Figure 3B). This reconstitution provided confidence that PKS12 protein could indeed synthesize mycoketide metabolite. For experimental ease, detailed characterization of products was performed with PKS12Δ1 protein.

In order to biochemically reconstitute the PKS12Δ1 protein, we coincubated the protein with different acyl-NAC starters (C_6_–C_18_) along with NADPH and radiolabeled MCoA. Alkaline hydrolysis followed by radio-TLC analysis revealed substrate specificity for short-/medium-chain starters (Figure 3C). PKS12Δ1 showed robust activity with the apparent *k*_cat_/*K*_M_ of 104.22 μM^−1^ sec^−1^ (Figure 3D). The activity was confirmed by constructing active site KS and AT null mutants, C2226A and the S2672A. Both these proteins were purified to homogeneity and were found to be catalytically inactive (Figure S3), thus confirming in vitro reconstitution of the PKS12Δ1 protein. A 2.5-fold improved catalytic activity of PKS12Δ1 along with better protein expression provided a good system to delineate the mechanisms involved in mycoketide formation.

**Figure 4:**
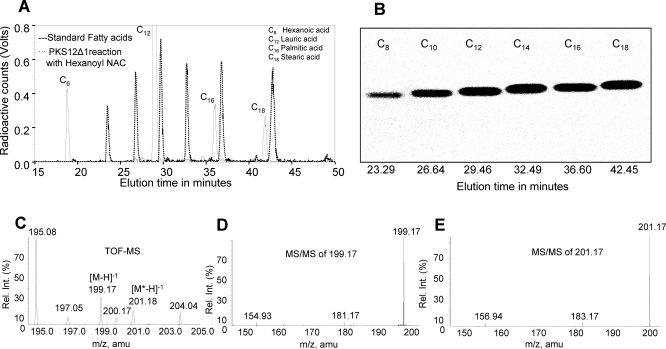
Radio-HPLC and Mass Spectrometry Characterization of PKS12Δ1 Products (A) Characterization of products on HPLC. The radioactive product synthesized by PKS12Δ1 was analyzed on reverse-phase HPLC, and the elution times were determined by a radioactive detector. The reaction was primed with a hexanoyl starter that undergoes two carbon extensions with MCoA. The product peaks superimpose with radioactive standards. The traces are stacked with an *x*-axis offset for clarity. (B) Autoradiograph of the HPLC-resolved PKS12Δ1 products that were concentrated and analyzed by radio-TLC. The *R*_f_ of various bands is C_8_ (0.468), C_10_ (0.476), C_12_ (0.492), C_14_ (0.507), C_16_ (0.523), and C_18_ (0.539). (C) ESI-MS analysis of the 29.46-min HPLC peak. Molecular ion peak of 199.17 confirms the eluent to be lauric acid. Isotopic distribution is in concordance with radioactive substrates utilized in the assay. (D) Tandem MS of [M-H]^−1^199.17 peak. The fragmentation pattern was similar to standard lauric acid corresponding to decarboxylation (154.93) and loss of water (181.17) from the parent ion. (E) Tandem MS of the [M^*^-H]^−1 14^C peak of lauric acid 201.17 peak. The characteristic peaks show mass difference of 2, corresponding to ^14^C radioisotope.

In order to unequivocally characterize the PKS12Δ1 product, we designed a radio high performance liquid chromatography (HPLC)-based assay, wherein fatty acids with different carbon chain lengths (C_3_ to C_18_) were separated on a reverse-phase column by using a ternary solvent system. Products formed from the PKS12Δ1 reactions primed with hexanoyl-NAC starter unit were first analyzed on TLC plates. The diffused product band was eluted and further subjected to HPLC analysis. Six radioactive products could be detected in the standard assay (Figure 4A). PKS12Δ1 protein is expected to add two carbon units during each cycle of chain extension. As can be observed from Figure 4A, three of these products showed identical retention times with standard radioactive fatty acids (C_12_, C_16_, and C_18_) (standard spectra shifted to the left for clarity). Other radioactive peaks, based on retention times and on our understanding of the programming of PKS enzymology, would correspond to C_8_, C_10_, and C_14_, respectively. Radio-TLC analysis of these HPLC-purified products showed a systematic change in *R*_f_ (Figure 4B), which could be unambiguously confirmed with their standard fatty acids. Mass spectrometric analysis of these peaks further confirmed the identity of products. Particularly in the case of longer chain products, the isotopic ^14^C peak was enhanced as compared to the corresponding ^13^C peak (Figure 4C). The electrospray tandem mass spectrometry (ESI-MS/MS) pattern for ^12^C and ^14^C peaks showed expected dehydration and decarboxylation peaks (Figure 4D and 4E). Based on these studies, we propose that the single-module construct of PKS12 protein can perform up to six rounds of chain extension in our in vitro enzymatic assays.

### Structural Analysis of PKS12 and N- and C-Terminus Linkers

**Figure 5:**
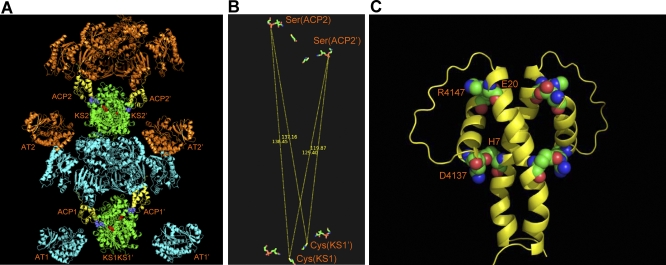
PKS12 Models and Identification of Interacting Residues (A) The end-to-end orientation of PKS12 is shown. Module 1 of PKS12 is shown in cyan and module two in copper brown, except the KS and ACP domains. The KS domains are shown in green and the ACP domains in yellow. (B) The active site residues of KS and ACP domains are shown in ball and stick, and the distances are shown. (C) Model for the docking domains of PKS12 generated by SCRWL and energy minimized using CVFF force field of Insight II. The interacting residues of the N- and the Cters are shown.

Our in vitro characterizations of PKS12 protein validates that this bimodular protein could indeed synthesize mycoketide product. An intriguing feature of the PKS12 catalysis would be the long-range transfer of ketide chain from module 2 to module 1. The recent structures of FAS [23] and KS-AT didomain from erythromycin PKS [24] provide an opportunity to investigate this possibility in the three-dimensional space. PKSs are proposed to be X-shaped homodimeric proteins, in which KS, DH, and ER provide the majority of the interfacial contacts in a given module. Modules that are present on two separate polypeptide chains interact through small helices. These helices protrude out from the N- and C-terminus ends to form an end-to-end organization [2,25,26]. The mammalian FAS structure also indicates similar end-to-end packing for consecutive modules in two neighboring unit cells along the common two-fold axis of the dimer [23]. This organization is likely to be conserved for modules, which are present on a single polypeptide chain [2]. Figure 5A shows the model of PKS12 generated by docking two KS-AT-DH-ER-KR-ACP modules in an end-to-end orientation. The template for KS-AT-DH-ER-KR has been taken from mammalian FAS structure, whereas the relative orientation of ACP with respect to KS has been fixed based on the ACP-KS interactions as seen in the yeast FAS structure [27]. Based on our model, the distances between the catalytic centers of ACP2 and KS1 are in the range of 130 Å (Figure 5B). Despite the inherent flexibility of the ACP domain, chain transfer over such a large distance seems unfeasible. This then raises a paradoxical problem for the iterative biosynthesis of mycoketide by PKS12.

Type I multifunctional PKSs synthesize polyketide products by either using an iterative mechanism with repetitive utilization of catalytic domains or by an assembly-line modular catalysis involving single use of active sites [28]. However, the programming that dictates these two mechanisms of biosynthesis has not been elucidated. Systematic analysis of various domains and linkers suggests an interesting comparison. The Cter modular proteins varies between 70–110 amino acids, whereas iterative linkers are typically smaller than 25 amino acids (G. Yadav and D. Mohanty; unpublished data). Moreover, a strong propensity to form helices could be observed in the case of modular linkers. A close examination of PKS12 protein suggested similarity to modular proteins, where the Cter is of 81 amino acids and also shows structural propensity to form three helices. This is in contrast to the Cters of two other mycobacterial iterative proteins, mycocerosic acid synthase (MAS) and PKS2 that have Cters of 11 residues and 13 residues, respectively, with no significant structural propensities. Furthermore, the analysis of the Nter of PKS12 also showed propensity to form a coiled-coil motif. Based on structural understanding of PKS proteins, the N- and C-termini cannot fold back to interact with one another. We therefore speculate that PKS12 protein molecules may form large supramolecular assemblies that interact with each other through the N- and C-terminus helices. We performed detailed analysis of PKS12 linkers to identify crucial residues involved in this docking complex. A chimeric model based on SCRWL [29] was built that utilizes the nuclear magnetic resonance (NMR) template of 6-deoxyerythronolide B synthase (DEBS) [25]. The residues in the helices of DEBS have been replaced by corresponding amino acids of PKS12, but the loops are retained (Figure 5C). The model was energy minimized using CVFF force field of Insight II [30]. Any two residues less than 5 Å apart were considered as interacting. This analysis led us to identify two key ionic interactions between C- and Nters (D4137 with H7 and R4147 with E20).

### Analytical Ultracentrifugation and Mutagenic Analysis of Interacting Linker Amino Acid Residues

**Figure 6:**
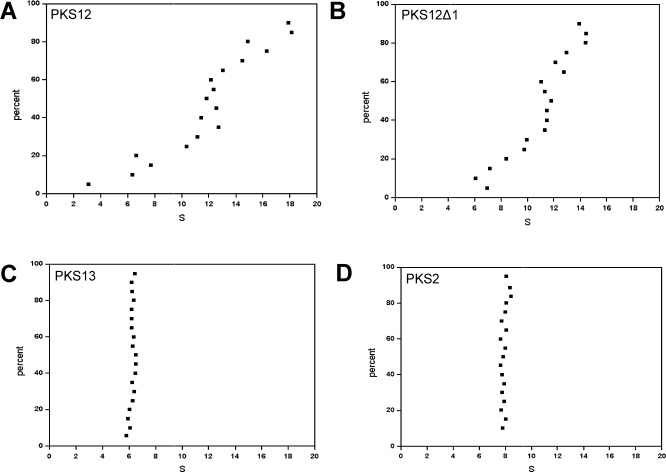
Sedimentation Velocity Analyses Analysis of PKS12 (A), PKS12Δ1 (B), PKS13 (C), and PKS2 (D) by sedimentation velocity in the XL1-analytical ultracentrifuge in 50 mM phosphate buffer (pH 7.2). The data were analyzed to yield the integral distribution of sedimentation coefficients.

In order to explore macromolecular organization of PKS12, we resorted to sedimentation velocity studies using the analytical ultracentrifuge. This analysis provides a sensitive measure to ascertain whether the sample consists of single or multiple species [31]. At low salt concentrations, both PKS12 and PKS12Δ1 showed a spread in the sedimenting boundaries (Figure 6A and 6B). These data could not be fitted to determine a unique sedimentation coefficient and suggested a possibility of multiple stable aggregation states. At the same time, we also performed control experiments with other mycobacterial PKS proteins. Both PKS13 and PKS2, which are single-module proteins with molecular weight of 186 and 225 kDa, respectively, showed a single sedimenting boundary (Figure 6C and 6D). The spread observed for PKS12Δ1 was also investigated at different protein concentrations, and a similar spreading pattern was observed in the sedimentation velocity profiles. These studies clearly suggested that PKS12 protein forms a large supramolecular assembly, probably through intermolecular interactions mediated by N- and C-termini helices. PKS proteins are known to posses weak intermolecular interactions, as has been demonstrated for DEBS [32] and Pps proteins (O.A. Trivedi and R. S. Gokhale, unpublished data) by using gel filtration chromatography. The number of species observed in the analytical ultracentrifugation studies could thus correspond to different states of oligomerization. Similar sedimenting profiles of intermolecular association has been previously observed for histone proteins [33].

Since intermolecular interactions in PKS12 protein is expected to be through the linker residues, we decided to completely swap the Nter residues with an N-terminus region from mycobacterial PpsA protein [20]. PpsA is the first module of the DIM biosynthetic machinery and contains an ACP loading domain at its N-terminus. This protein also does not show any coiled-coil region at its N-terminus end. This fusion construct of PKS12Δ1 showed extremely poor catalytic activity, thus emphasizing the significant role of these linker residues (Figure S3). We also performed analytical ultracentrifugation studies on this linker-change PKS12Δ1 (Figure S4). Interestingly, the scatter in sedimenting boundaries markedly observed for the wild-type protein was completely abolished. We then decided to carry out product analysis using radio-HPLC.

**Figure 7:**
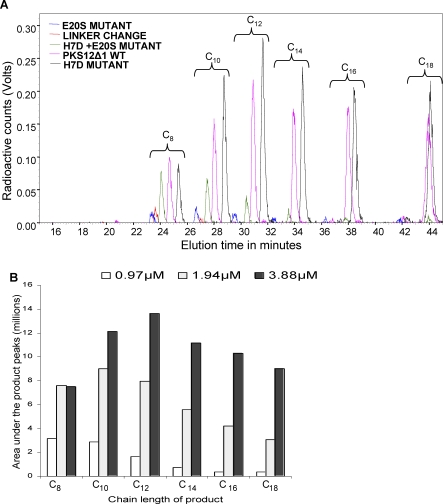
Quantitative Radio-HPLC Analyses of Linker-Change and Single Amino Acid Linker Mutants of PKS12Δ1 and Effect of Concentration on PKS12Δ1 Catalysis (A) The radioactive products synthesized by PKS12Δ1 and PKS12Δ1 mutant proteins were analyzed on reverse-phase HPLC, and the elution times were determined by using a radioactive detector. All reactions were primed by hexanoyl starter that undergoes two carbon extension(s) with MCoA. Trace for E20S mutant (blue) shows exact retention time while traces of all other mutant proteins are stacked with an *x*-axis offset for clarity; linker-change protein (red), H7D + E20S mutant (green), wild-type PKS12Δ1 (pink), and H7D mutant (black). Chain lengths of standard fatty acids eluting at these retention times are marked on these stacked traces. Value of 0.01 V is considered as background, based on the control experiments. (B) Comparison of concentration dependent changes in the product profiles catalyzed by PKS12Δ1. Gradation in color indicates increase of protein concentration for each set of peaks, which in turn depict the product formed. At higher protein concentration, more of longer chain length products are synthesized, since there is a shift in equilibrium towards higher order multimeric species.

Analysis of the linker-change protein indicated that this protein could carry out only one round of malonyl-CoA condensation to synthesize C8 fatty acid (red trace in Figure 7). Clearly, this linker-change has abrogated the ability of PKS12Δ1 to perform iterations through formation of higher order oligomers. In order to directly explore the role of PKS12 linker residues, we decided to make site-specific mutants. Based on our PKS12 docking domain model (Figure 5C), we predicted a strong ionic interaction between E20 from N-terminus with C-terminus R4147 residue. The PKS12Δ1 E20S mutant protein showed a 5-fold decrease in its catalytic turnover. Quantitative radio-HPLC analysis also showed greater than 5-fold decrease in its product formation (blue trace in Figure 7). This compromised activity resulting from a change in one amino acid in its linker provides strong evidence of their requirement in efficient catalysis. H7D mutation remarkably led to enhancement of catalytic activity (black trace in Figure 7). Although the exact reason for this behavior is not clear, our computational analysis suggests that such a modification could provide favorable intrahelical contacts. Moreover, sequence alignment in this region is rather poor, and minor structural alterations can clearly provide a stabilizing effect. A double mutant constructed by combining these two mutations clearly showed an additive effect (green trace in Figure 7). The product profile was somewhat an average of two independent mutations. It is interesting to note that products formed from higher iterations were significantly lower in comparison to wild-type and H7D proteins. Clearly, single amino acid substitutions in the noncatalytic regions show effect on the catalytic rates of product formation, thus reemphasizing their importance in mycoketide biosynthesis through intermolecular iterative catalysis.

### Protein Concentration-Dependent Changes in PKS12Δ1 Product Profile

Since intermolecular interactions show dependence on concentration, we examined the formation of products by PKS12Δ1 at different protein concentrations. For an intermolecular iterative catalysis, the formation of the final product would be dependent on the number of protein molecules participating in the multimeric assembly. We therefore hypothesized that formation of long-chain products would be facilitated at higher concentrations, whereas at lower concentration, the product profile should support biosynthesis of short-chain fatty acids. We investigated the product profile using radio-HPLC at three different concentrations of 0.97 μM, 1.94 μM, and 3.88 μM by using hexanoyl-NAC as the starter unit. As is evident from Figure 7B, the maximum amount of product formed at the lowest concentration corresponds to a single round of condensation of C2 unit. Moreover, all subsequent products are produced at lower amounts than the C8 product. At the highest protein concentration, there is favorable equilibrium towards formation of higher order multimeric species, and as can be observed from Figure 7B, there is a clear shift in product profile pattern, and C8 is the lowest of all the products. Such an effect would not be observed for proteins that would use an intramolecular iterative mode of catalysis.

### Specific Inhibition of Catalysis by C-Terminus Linker Peptide

**Figure 8:**
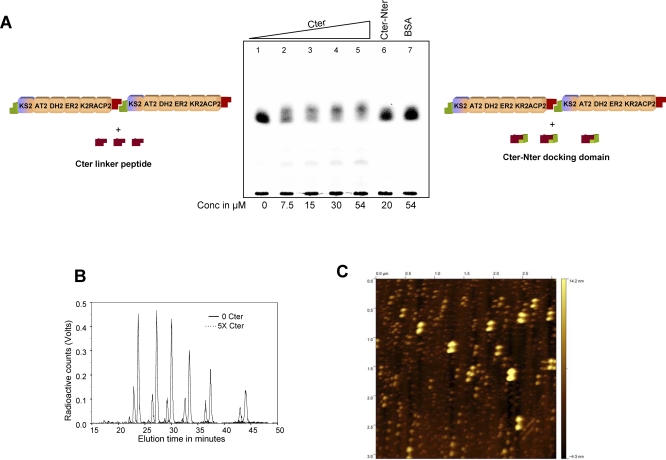
Effect of Cter Peptide on PKS12Δ1 Catalysis and AFM Analysis of PKS12Δ1 (A) Effect of Cter peptide, Cter-Nter concatenated peptide, and BSA on PKS12Δ1 catalysis. Specific inhibition of enzymatic activity by C-ter peptide can be seen from lanes 1 to 5. C-terminus peptide competes with other PKS12Δ1 molecules and thus specifically inhibits product formation. BSA and the concatenated C- and N-ter linker, which in itself forms a stable docking complex, does not interfere with PKS12Δ1 catalysis. (B) Radio-HPLC analysis of PKS12Δ1 reaction inhibited by five times molar excess of Cter peptide. The solid curve indicates the control run without any Cter peptide, and the dotted curve shows the profile for inhibited assay. (C) AFM analysis of PKS12Δ1 showing multimeric organization.

Since the supramolecular organization of PKS12 protein is proposed to be through N- and Cters, we wanted to investigate whether linker peptides would disrupt protein-protein interactions and thus affect catalysis. We therefore decided to assay PKS12Δ1 protein in presence of varying concentrations of purified Cter peptide. Towards this end, we cloned and expressed Cter peptide of PKS12 protein in BL-21 strain of *E. coli* by using T7 expression vector. Purification by Ni^2+^-NTA chromatography followed by anion exchange chromatography yielded PKS12 Cter peptide at high purity. Coincubation of this peptide with PKS12Δ1 protein showed a clear decrease in the formation of enzymatic products (Figure 8A, lanes 1–5). This was further confirmed and quantitated by radio-HPLC analysis of PKS12Δ1 reactions (Figure 8B), in which a marked change in product profile was observed. As a control, an equivalent concentration of bovine serum albumin protein was also used in the assay, which showed no effect on the enzymatic activity (Figure 8A, lane 7).

The specificity of inhibition by Cter peptide was further investigated by using a covalently concatenated Cter–Nter peptide of PKS12. Such a Cter-Nter fusion construct was previously used for structural elucidation of docking domain in erythromycin polyketide synthases, in which these linkers form a stable docking complex by forming a pseudo four-helix bundle [25]. The Cter-Nter peptide was expressed in E. coli by splicing Nter at the end of the Cter sequence. The propensity of Cter and Cter-Nter peptides to adopt helical conformations was investigated by using circular dichroism (CD). The ratio of the negative molar ellipticities at 220 and 208 nm is 0.65 for Cter peptide and 1.08 for Cter-Nter peptide. Ratio of approximately 1.0 has been suggested to be typical of interacting α-helices [34,35]. Taken together, these CD studies suggest that Cter peptide forms α-helices, which together with Nter peptide forms an interacting pseudo four-helix bundle. Enzymatic assays performed in the presence of Cter-Nter peptide showed no inhibition of PKS12Δ1 catalytic function (Figure 8A, lane 6). These studies thus demonstrate that Cter peptide specifically perturbs PKS12Δ1 oligomerization by competitively interacting with the Nter region of PKS12Δ1 protein.

### Atomic Force Microscopy Analysis of PKS12Δ1

Multimeric organization of PKS12 protein was further validated by performing atomic force microscopy studies. PKS12Δ1 protein from the assay mixture was used for visualization under noncontact acoustic AC mode. Figure 8C shows the presence of singular particles and some linear oligomers randomly distributed over the entire surface. Singular particle dimensions as analyzed by SPIP software could correspond to native monomers of PKS12Δ1. Interestingly, a distinctive pattern of linear multimeric species of varying length could also be observed. Using the height scaling method for these measurements, the linear multimeric species are not loosely interacting groups of single particles, instead, are professedly to be unified entities. These studies reinforce association between protein molecules.

### Intermolecular Acyl Chain Transfer between Two Proteins

**Figure 9:**
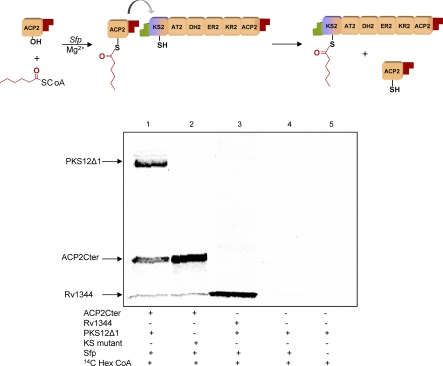
ACP to PKS Transfer Assays Sfp from B. subtilis was used to label ACP2Cter of PKS12 (lane 1 and 2) and a noncognate ACP protein, Rv1344, from M. tuberculosis with ^14^C hexanoyl CoA. Although ACP2Cter was able to transfer the label to PKS12Δ1 (lane 1), no transfer was seen when either the KS mutant of PKS12Δ1 was used (lane 2) or when a noncognate ACP with PKS12Δ1 was used (lane 3). Minus ACP control experiment did not show any label on PKS12 (lane 4); there was also no direct labeling of PKS12Δ1 observed (lane 5)

The unambiguous proof of intermolecular iterative mechanism of biosynthesis for mycoketide was established by designing an assay in which direct transfer of acyl chain from ACP of one protein molecule onto the KS domain of another PKS protein molecule could be demonstrated. Towards this end, we cloned and purified the ACP2 domain along with the Cter (ACP2Cter) of PKS12. The ACP2Cter protein was expressed in the apo-form and then Sfp protein was used to directly load ^14^C-hexanoyl phosphopantetheine group onto the catalytic Ser of ACP [36]. Coincubation of this protein with PKS12Δ1 showed facile transfer of radioactivity to this large PKS protein (Figure 9, lane 1). This transfer was not observed for the KS active site mutant protein of PKS12Δ1 (C2226A) (Figure 9, lane 2). The specificity of the interactions was further investigated by using another ACP protein (Rv1344) from M. tuberculosis. Although the Rv1344 protein could be acylated with ^14^C-hexanoyl phosphopantetheine group, no transfer could be seen on incubation with PKS12Δ1 (Figure 9, lane 3). Appropriate control experiments showed no direct labeling of PKS12Δ1, both in the presence or absence of Sfp protein (Figure 9, lanes 4 and 5). Taken together, these studies explicitly demonstrate specificity of intermolecular interactions and also of transfer of acyl chain from the phosphopantetheine group of ACP to the catalytic Cys residue of the KS domain.

## Discussion

This study unravels a novel biosynthetic mechanism by which mycobacterial PKS12 protein is involved in the biosynthesis of the acyl chain of MPMs. Cell-free reconstitution of a 431-kDa PKS12 protein along with systematic biochemical analysis has facilitated characterization of a unique supramolecular organization. We show that this assembly is mediated through precise intermolecular interactions between its N- and Cters in a head-to-tail association. The organization of this supramolecular assembly could be explained based on the recent three-dimensional structures of FASs [23] and the high resolution structure of KS-AT didomain from module 5 of erythromycin PKS [24]. The overall X-shaped dimeric structure of FAS is built around KS dimer interface. DH and ER domains also provide stability to this homodimeric protein, whereas AT and KR catalytic domains are located away from the dimer interface. It is clear from these structures that the ACP domain undergoes large conformational changes. The phosphopantetheine arm on ACP is expected to reach out to various domains within the same module and also to the KS domain of the next module to facilitate catalysis in these modular proteins [26].

The noncovalently associated cognate modules have been proposed to interact through C- and N-terminus helices that form an independent docking complex [21,25]. As demonstrated in the DEBS KS-AT structure, the N-terminus helix protrudes away from the KS dimer interface and forms a stable four-helix bundle with C-terminus helices of the previous module. The two homodimeric modules thus are arranged in an end-to-end fashion [26]. Since positions of modules could be changed by suitable engineering of linkers, the organization of two covalently linked contiguous modules is expected to follow similar organization [2,21]. PKS12 protein, investigated in this study, possesses a bimodular organization, which was proposed earlier to catalyze five rounds of alternative condensations of methylmalonyl and malonyl units through an iterative mechanism. Such a biosynthetic route would require transfer of intermediate chain from ACP of module 2 to KS of module 1. Based on present understanding of modular assembly, this chain transfer seems unfeasible. Therefore, in this study, we have systematically dissected programming of PKS12 to biosynthesize mycoketide.

In recent years, “stuttering” as well as “skipping” of modules has been reported during biosynthesis of polyketides that deviate from the linear processive mechanistic logic of modular biosynthesis [37–39]. The modular PKSs of borrelidin and aureothin are two recent examples that illustrate a controlled iterative utilization of selective modules during polyketide biosynthesis [40,41]. We therefore first confirmed the specificity of AT domains from two modules of PKS12 to confirm that the two modules are used alternately during mycoketide biosynthesis. This experimental confirmation is in agreement with our previous studies, in which we had demonstrated that a crucial Phe residue acts like a gatekeeper that discriminates between malonate and methyl malonate specificity [42]. The module 2 of PKS12 protein indeed possesses this important Phe residue that restricts its specificity to malonyl extender unit. The corresponding amino acid in module 1 is Ser and therefore is able to utilize methylmalonyl extender units.

In vitro enzymatic assays for PKS12 and PKS12Δ1 confirmed that this protein could indeed synthesize mycoketide. Detailed analysis of the N-ter and C-ter sequences suggested a modular mode of catalysis for PKS12. The organization of PKS12 proteins to form a larger molecular assembly was demonstrated by analytical ultracentrifugation. The significance of this organization was investigated by replacing N-ter and also by site-specific mutagenesis of crucial amino acids. Although the linker-change protein led to a single round of chain extension, the single amino acid mutants showed appreciable change in overall catalysis. Similar studies earlier with DEBS protein has shown crucial role of single amino acids in forming a docking complex [43]. More evidence for intermolecular iterative catalysis was provided by investigating the concentration-dependent changes in the product profiles of PKS12Δ1. At higher protein concentration, more of longer chain products are synthesized, since there is a shift in equilibrium towards higher order multimeric species. Furthermore, the Cter showed specific inhibition of enzymatic activity by disrupting protein–protein interactions while competing for the cognate N-ter of PKS12 protein. Quite remarkably, the docking domain consisting of Cter-Nter concatenated protein does not inhibit catalysis. CD analysis of these linker peptides supports the formation of docking domains through interacting helices. The imaging studies of PKS12Δ1 performed by using AFM also clearly showed propensity to form oligomeric structures. An unambiguous proof of this novel organization comes from our experiment in which we demonstrate specific transfer of acyl chain from (*n* − 1) protein to the next protein (*n*), which are designed to specifically interact through C- and Nters, respectively.

**Figure 10:**
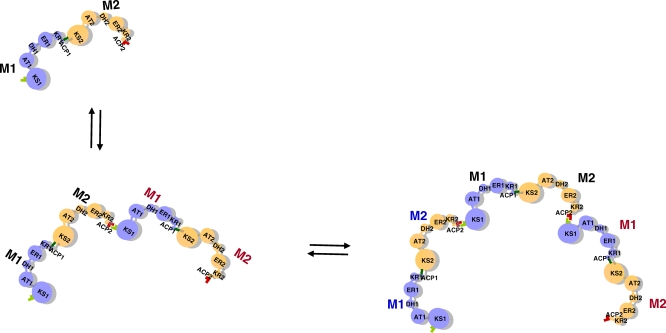
Schematic Representation of Modularly Iterative Catalysis Supramolecular organization of PKS12 protein formed by interactions between N- and Cters.

PKS12 protein thus forms a modular assembly to perform repetitive condensation analogous to iterative proteins. We therefore refer this mechanism of catalysis as “modularly iterative” (Figure 10). To the best of our knowledge, this is probably the first example wherein intermolecular iterative catalysis has been characterized by forming a novel structural organization. Interestingly, two recent studies have provided evidence for the formation of a large, singular mega-complex involving several PKS and FAS proteins [44,45].

Biosynthesis of MPMs would require other downstream enzymes that would release and phosphorylate the nascent acyl chain and then carry out final mannosylation. The computational analysis of this region in the Mtb genome suggests few putative enzymes that could be involved in the complete biosynthesis of MPMs. Associated with this cluster is LipT, possessing an esterase motif, which could release the mycoketide from PKS12, similar to the lovD protein from lovastatin biosynthesis [11]. Alternatively, Rv2047c could reductively release alkyl chain from PKS12 protein. Rv2047c protein shows homology with recently characterized reductive domains from multifunctional non-ribosomal peptide synthetases. This protein also contains a swivel domain commonly found in phosphoenol pyruvate (PEP)-utilizing enzymes that may be required for phosphorylation. Located upstream of PKS12 is Ppm1, encoding a polyprenol monophosphomannose synthase that could mannosylate the phophorylated alkyl chain [46].

The novel intermolecular iterative biosynthetic mechanism identified in this study provides new perspective to our understanding of polyketide biosynthetic machinery. This study also highlights clever ways by which mycobacteria utilize polyketide biosynthetic machinery to produce exotic lipid metabolites.

## Materials and Methods

The bacterial artificial chromosome (BAC) library of M. tuberculosis H37Rv was obtained from the Pasteur Research Institute, France. Radioactive malonyl CoA were purchased from Perkin Elmer Life Sciences (1.9166 GBq/mmol) and radioactive methylmalonyl CoA was purchased from American Radiolabeled Chemicals (55 mCi/mmol). E. coli XL1-blue and E. coli BL21 (DE3) were used as cloning and expression strains, respectively. All other chemicals used were of analytical grade.

### Cloning of *pks12* and and *pks12* constructs.

BAC genomic DNA of M. tuberculosis H37Rv [47] was digested with restriction enzyme NheI, and the fragments were cloned in a NheI-digested vector, pTC1. Cloning vector pTC1 has NdeI to HindIII multiple cloning site region of pET28c inserted in the EcoRV site of pBluescript-SK+ (pBS) vector. NheI site was specifically utilized to obtain a 14,412-bp genomic DNA fragment, which includes the complete *pks12* gene. Gene *pks12* was cloned in two parts from pTC2 which contains this 14,412-bp fragment. In brief, the N-terminus 827 bp of *pks12* was amplified (primers RSG675 to RSG678) by overlap extension PCR while inactivating an NdeI (632 bp) site via silent mutations to facilitate cloning. This NdeI (1 bp) to PstI (827 bp) fragment was cloned in the SnaBI site of pLITMUS 28, and the construct was further used to insert a PstI (827bp)–PstI (6,896 bp) fragment from pTC2 to yield pTC5. The C-terminus 1,379-bp fragment was PCR amplified using primers RSG679 and RSG681. The AscI (11,077 bp)–HindIII (12,456 bp) fragment was cloned in pNEB193 followed by insertion of NdeI (6,701 bp) to AscI (11,077 bp) fragment from pTC2. This NdeI (6,701 bp) to HindIII (12,456 bp) fragment was inserted in pET21c (+) followed by insertion of NdeI (1 bp)–NdeI (6,701 bp) from pTC5 to create PTC10, the expression plasmid for PKS12 with a C-terminal polyhistidine tag. Primer sequences used for cloning are given in Table S2.

*pks12Δ1* was constructed by deleting BsaBI (112 bp)–BsaBI (6,178 bp) fragment from pTC5. The clone thus obtained was called pTC13. pTC13 was utilized to insert the NdeI (1 bp) to NdeI (6,701 bp) fragment containing this deletion alongside NdeI (6,701 bp) to HindIII (12,456 bp) fragment in pET21c (+). The clone obtained was called pTC15 and was used for the purification of PKS12Δ1.

The linker-change mutant of *pks12* was constructed by replacing the N-terminal NdeI to BsaBI fragment of *pks12* with the N-terminal NdeI to BsaBI fragment of *ppsA* gene by using the conserved BsaBI site present at the start of the KS domain. Although BsaBI site was already present in *pks12*, it was engineered in *ppsA* via silent mutations.

For construction of the PKS12 Cter peptide, the C-terminus 243 bp was PCR amplified (primers RSG1505, RSG1506) from pTC2 while engineering restriction sites at the terminal ends to facilitate cloning. For concatenated Cter-Nter fusion construct, Cter and Nter were separately PCR amplified (primers RSG1426, RSG1427 for Cter and RSG1428, RSG1430 for Nter) while engineering a PstI site at the end of Cter and PstI site at the beginning of Nter to facilitate their joining.

AT2 null mutant of PKS12 was constructed by insertion of NdeI (1 bp)–NdeI (6,701 bp) from pTC5 in NdeI-digested AT mutant of PKS12Δ1, pTC23.

ACP2Cter was PCR amplified (primers RSG 1718, RSG 681) and cloned in pET21c (+) expression vector.

### Site-directed mutagenesis.

Site-directed mutagenesis was used to generate the point mutants of *pks12Δ1*. The mutations are shown in Table S1, and the mutagenic primers are shown in Table S2. To minimize errors, small fragments containing the region of interest were subcloned and inserted back after mutations were introduced. All mutants were constructed by using Quickchange Site Directed Mutagenesis Kit (Stratagene). Mutations were confirmed by restriction analysis and by DNA sequencing.

### Expression and purification of proteins.

All proteins were expressed and purified using the following procedure. The expression plasmids were cotransformed in BL21-DE3 strain of *E .coli* with pRSG56 to enable coexpression of Sfp for phosphopantetheinylation of the ACP domain wherever required. The cells were grown at 30 °C in LB medium with 50 μg ml^−1^ of antibiotic (carbenicillin/kanamycin) to an absorbance of 0.6 at 600 nm. The cells were incubated on ice for 10–15 min, and induced with 0.1 mM to 1 mM isopropyl thio–β-D-galactoside for 12–18 h at an appropriate temperature (22 °C to 37 °C). The cells were harvested by centrifugation (3,500 *g*, 10 min; Beckman Coulter Allegra 6R), resuspended in buffer A (50 mM Tris chloride [pH 8.0] containing 10% glycerol) containing 150 mM NaCl, lysed using a French press, and cellular debris was removed by centrifugation (24,000 rpm, 40 min; Sorvall evolution RC, rotor: SA 300). Polyethylene imine was added to a final percentage of 0.1%, and DNA pellet was removed by centrifugation (20,000 rpm, 20 min; Sorvall evolution RC, rotor: SA 300). Ni^2+^-nitrilotriacetic agarose resin was added to the supernatant (0.75 ml l^−1^ of culture), and the proteins were purified using affinity chromatography with increasing concentration of imidazole in buffer A. Anion exchange chromatography was performed by using a 6-ml Resource-Q column and AKTA chromatography system (Amersham). The protein was purified in buffer B (100 mM phosphate buffer [pH 7.2] containing 10% glycerol) and eluted with an increasing gradient of 1 M NaCl in buffer B with a flow rate of 2 ml min^−1^.

ACP2Cter expressed as inclusion bodies at all temperature and IPTG concentrations tested. After French press, the pellet containing inclusion bodies was resuspended in buffer containing 2 M Urea in 100 mM phosphate buffer (pH 7.2), 10 mM Tris buffer (pH 8.0) and 1 mM DTT. Supernatant was separated from the 2 M pellet by centrifugation (15,000 rpm, 30 min), the pellet was resuspended in a higher concentration of urea until the concentration reached 8 M, and supernatant was separated at each stage. SDS-PAGE analysis of all the fractions showed presence of ACP2Cter in 2 M fractions. This suspension was subjected to a 100-fold “snap-dilution” in the refolding buffer containing 50 mM Tris, 10% Glycerol and 150 mM NaCl. The refolded protein was purified and used for transfer assays.

Protein concentrations were determined by using BCA protein estimation kit (Pierce).

### Labeling of enzymes by ^14^C-methylmalonyl- and ^14^C-malonyl-CoA.

Enzymes (1–5 μM) were incubated with ^14^C-MCoA and ^14^C-MMCoA (36 μM) at 4 °C for 2 min. The reactions were quenched by addition of SDS-PAGE loading dye lacking any reducing agents like dithiothreitol or β-mercaptoethanol. Samples were directly loaded on a 6% SDS-PAGE gel, and electrophoresis was performed at 25 mA until the dye front ran out. The gel was dried and analyzed using a phosphorImager (Fuji BAS5001).

### Product formation assays.

PKS12 enzymatic assays included 100 mM phosphate buffer (pH 7.2), 150 μM acyl-NACs, 75 μM MCoA, 75 μM MMCoA, 7.2 μM ^14^C-MCoA, 7.2 μM ^14^C-MMCoA, 4 mM NADPH, 10% glycerol, 2 mM Tris (2-carboxy ethyl) phosphine hydrochloride (TCEP), and 2.5–4.5 μM protein in 100-μl reaction volume. The reactions were incubated at 30 °C for 6–12 h. The products were released with 45% KOH followed by acidification and extraction in hexanes or ethyl acetate. The extract was spotted on TLC plate that was developed by using 40:60:1 hexanes:ether:acetic acid. The radio-TLC plates were analyzed by using a phosphorimager. For PKS12Δ1, the enzymatic assays included all the components except MMCoA. For radio-HPLC assays, the concentration of ^14^C-MCoA was increased to 25 μM while keeping the total MCoA concentration as 100 μM. The band of interest was scraped from TLC, extracted in ethyl acetate, concentrated and injected on C18 reverse-phase column (gradient: 100% B in 20 min, 100% B in 40 min, 20% B in 60 min; A: H_2_O, B: 5% methanol in ACN with 0.1% formic acid) on HPLC (Shimadzu). To analyze the HPLC eluted peaks, PKS12Δ1 products were first run on TLC plates, these were then extracted and separated on HPLC and then further analyzed on TLC plates. The radioactivity was monitored online by a radioactive detector (IN/US system β-RAM Model 3). For kinetic analysis, radiolabeled products were quantified using PhosphorImager (Fuji BAS5001).

For competition experiments, the competing proteins (Cter, Cter-Nter, and BSA) were titrated into reaction mixtures containing 100 mM phosphate buffer (pH 7.2), 75 μM MCoA, 7.2 μM ^14^C-MCoA, 4 mM NADPH, 10% glycerol, 2 mM TCEP, and 2.5 μM protein in 100-μl reaction volume. The reactions were analyzed in a manner similar to PKS12Δ1 assays.

### ACP to PKS transfer assays.

The ACP to PKS transfer assays were carried out in two steps wherein first the ACP protein was exogenously labeled with ^14^C Hexanoyl CoA in a reaction containing 45 μM ^14^C Hexanoyl CoA, 12.5 mM MgCl_2,_ and the Bacillus subtilis enzyme Sfp [48]. The reaction was carried out for 15 min at 37 °C, quenched with 0.05 M EDTA, and PKS proteins were added to a final concentration of 1.5 to 2 μM. The reaction mixture was incubated for 15 min at 30 °C for the transfer to occur. The reactions were quenched by addition of SDS-PAGE loading dye lacking any reducing agents, such as dithiothreitol or β-mercaptoethanol. Samples were directly loaded onto a 10% SDS-PAGE gel, and electrophoresis was performed at 25 mA. The gel was dried and analyzed using a Phosphorimager (Fuji BAS5001).

### Synthesis of alkyl NAC thioesters.

Various alkyl NAC thioesters and 9-hydroxy decanoyl NAC was synthesized according to a modified protocol reported earlier [20,49]

### Sequence analysis and structural modeling.

Primary sequence of the gene was obtained from Tuberculist (http://genolist.pasteur.fr/TubercuList/). Secondary structure analysis and multiple sequence alignment for the PKS12 chimera model were carried out by using seqweb software. Domain boundaries for PKS12 were obtained from SEARCHPKS, a program developed for detection and analysis of PKS domains (http://rd.plos.org/pbio.0060163). The chimera model for linkers was built using SCRWL [29] in which the intervening loops of the DEBS docking domains were kept and only the interacting helices were changed to identify putative interacting residues. The model for bimodular PKS12 was generated by docking KS-AT segment of module 2 with DH-ER-KR segment of module1 using the software 3D-DOCK [50]

### Sedimentation velocity analysis.

Sedimentation velocity experiments were conducted with an optima XLI instrument (Beckman Coulter) using an An 60Ti four-hole rotor at 35,000 rpm at 20 °C with three 420-μl samples in standard double-sector Epon centerpieces equipped with quartz windows. The sample A_280_ ranged from 0.6–0.9. Sedimentation velocity data were collected using continuous scan mode set at 0.001-cm radial increments. The scans were analyzed by the method of Van Holde-Weischet [51]. These data yielded the integral distribution of sedimentation coefficients plotted as the boundary fraction versus S_20,w_ (sedimentation coefficient corrected for water at 20 °C )

### Atomic force microscopy.

Pico plus atomic force microscope (Agilent Technologies) was used in acoustic AC (noncontact) mode for imaging. Images were recorded in air with either a bare mica surface or mica with the samples (normal spring constant of cantilever: 2.8 N/m, frequency: 59.722 kHz). The PKS12Δ1 reaction mixture was diluted 20-fold with 100 mM phosphate buffer and was immobilized on freshly cleaved mica for 30 s. The sample was washed with nanopure water, dried under N_2_, and subjected to AFM analysis.

### Circular dichroism analysis.

CD spectra were recorded on a Jasco-710 instrument equipped with a temperature cell holder. The spectra were an average of ten scans, and the data were presented as mean residual ellipticity (MRE) expressed in deg cm^2^ dmol^−1^. The protein concentrations were determined by using BCA protein estimation kit (Pierce).

## Supporting Information

Figure S1 PKS12 Activity in Absence of Any of the Two Extenders PKS12 protein was assayed for activity as given in Materials and Methods. While PKS12 was active in presence of both extenders (lane 1), absence of MCoA (lane 3), or MMCoA (lane 2) from the assays rendered the protein inactive.
(70 KB PDF)

Figure S2 Effect of KS Inhibitor, Cerulenin, and Heat Inactivation on Enzymatic Assays of PKS12 PKS12 protein (lane 1), PKS12 protein with 100 μM cerulenin (lane 2), and PKS12 protein with ethanol equivalent to the volume of cerulenin added (lane 3) were incubated at 30 °C for 1 h prior to setting up assays as given in Materials and Methods. Extractions were done in hexanes to extract only the polyketide product. Cerulenin renders the KS domain inactive by forming a covalent adduct and inhibits the reaction (lane 2). Preincubation (lane 1) and ethanol (lane 3) do not lead to any inhibition. PKS12 protein activity was further confirmed by denaturing the protein by boiling at 100 °C for 10 min and setting up assays (lane 4).
(63 KB PDF)

Figure S3 PKS12Δ1, KS Mutant, AT Mutant, and Linker-Change Protein Activity Assays PKS12Δ1, KS mutant (C2226A), AT mutant (S2672A), and linker-change protein were assayed for activity as given in Materials and Methods. Although PKS12Δ1 showed robust activity (lane 1), KS mutant (lane 3) and AT mutant (lane 4) are totally inactive. Lane 5 shows the activity assays for the linker-change protein, and no background activity is seen in the absence of protein (lane 2).
(69 KB PDF)

Figure S4 Analytical Ultracentrifugation Analysis of Linker-Change Mutant Analysis of linker-change mutant of PKS12Δ1 by sedimentation velocity in the XL1-analytical ultracentrifuge in 50 mM phosphate buffer (pH 7.2). The data were analyzed to yield the integral distribution of sedimentation coefficients.
(13 KB PDF)

Table S1 Highlighting Mutations for Site-Directed Mutagenesis of Key Residues Mutations are indicated in bold
(11 KB PDF)

Table S2 List of Primers Used (8 KB PDF)

## Abbreviations

ACP: acyl carrier protein
AFM: atomic force microscopy
AT: acyl transferase
CD: circular dichroism
Cter: C-terminus linker
DH: dehydratase
ER: enoyl reductase
FAS: fatty acid synthase
HPLC: high performance liquid chromatography
KR: ketoreductase
KS: ketosynthase
MCoA: malonyl coenzyme A
MMCoA: methylmalonyl coenzyme A
Nter: N-terminus linker
PKS: polyketide synthase
PKS12Δ1: module 1-deleted polyketide synthase 12 protein
Sfp: surfactin phosphopantetheinyl transferase
TLC: thin layer chromatography
