# Supplementary material for: Novel Intermolecular Iterative Mechanism for Biosynthesis of Mycoketide Catalyzed by a Bimodular Polyketide Synthase
Source: PLoS Biol. 2008 Jul 8;6(7):e163. doi: 10.1371/journal.pbio.0060163 (PMC2443190; doi:10.1371/journal.pbio.0060163)
Supplement: Supplementary file 1 [file pbio.0060163.sg001.pdf]

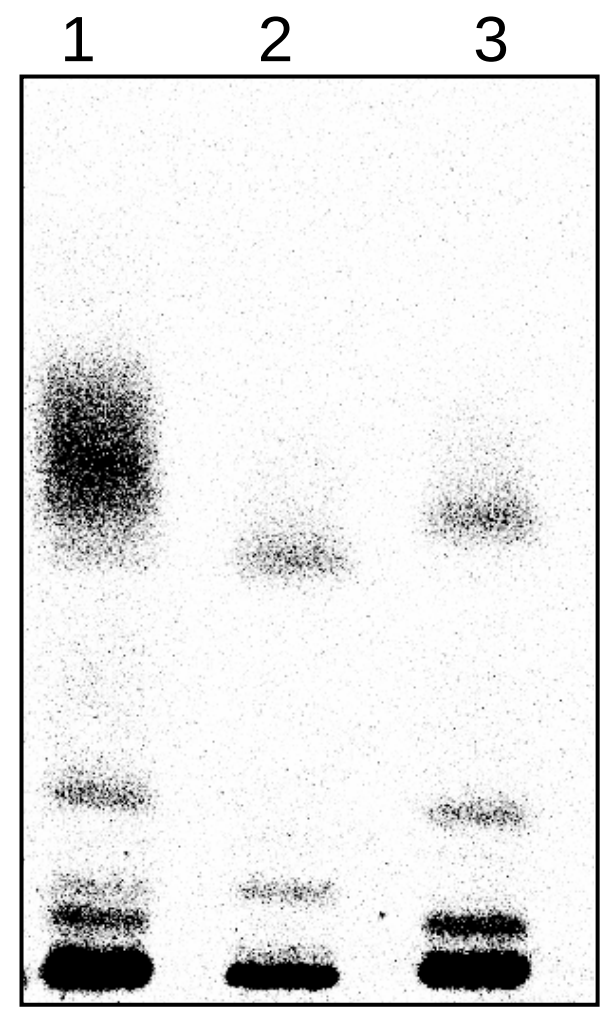

	1	2	3
**PKS12**	+	+	+
**HEX SNAC**	+	+	+
**MCoA**	+	+	-
**MMCoA**	+	-	+
