# Supplementary material for: Novel Intermolecular Iterative Mechanism for Biosynthesis of Mycoketide Catalyzed by a Bimodular Polyketide Synthase
Source: PLoS Biol. 2008 Jul 8;6(7):e163. doi: 10.1371/journal.pbio.0060163 (PMC2443190; doi:10.1371/journal.pbio.0060163)
Supplement: Supplementary file 3 [file pbio.0060163.sg003.pdf]

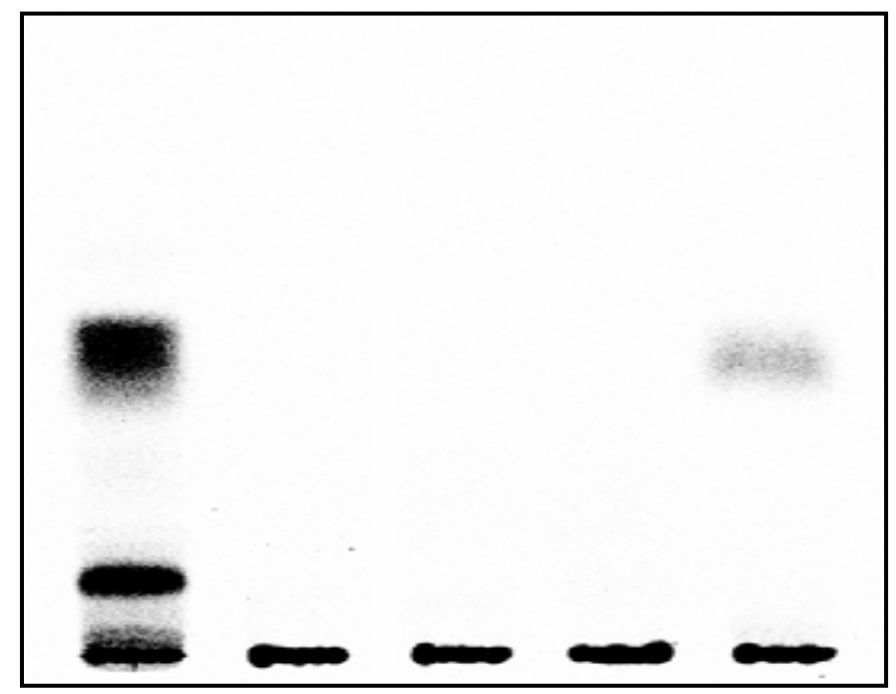

	1	2	3	4	5
**PKS12Δ1**	+	-	-	-	-
**PKS12Δ1 - KS mutant**	-	-	+	-	-
**PKS12Δ1 - AT mutant**	-	-	-	+	-
**PKS12Δ1 - Linker swap**	-	-	-	-	+
