# Supplementary material for: Novel Intermolecular Iterative Mechanism for Biosynthesis of Mycoketide Catalyzed by a Bimodular Polyketide Synthase
Source: PLoS Biol. 2008 Jul 8;6(7):e163. doi: 10.1371/journal.pbio.0060163 (PMC2443190; doi:10.1371/journal.pbio.0060163)
Supplement: Supplementary file 4 [file pbio.0060163.sg004.pdf]

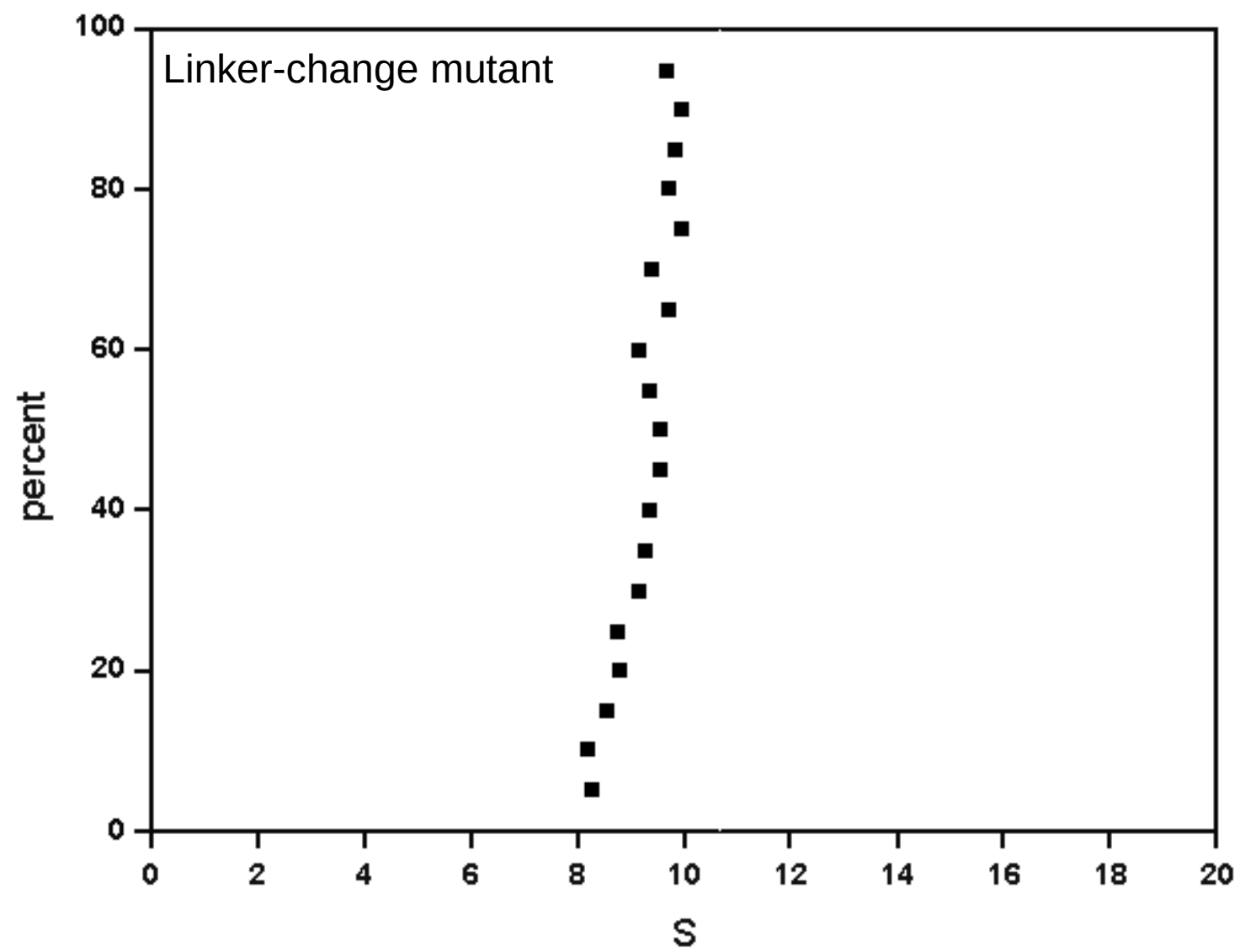

Linker-change mutant
percent
S
0
20
40
60
80
100
0
2
4
6
8
10
12
14
16
18
20
