# Supplementary material for: Novel Intermolecular Iterative Mechanism for Biosynthesis of Mycoketide Catalyzed by a Bimodular Polyketide Synthase
Source: PLoS Biol. 2008 Jul 8;6(7):e163. doi: 10.1371/journal.pbio.0060163 (PMC2443190; doi:10.1371/journal.pbio.0060163)
Supplement: Supplementary file 5 [file pbio.0060163.st001.pdf]

1. KS mutant at C2226A (Primers: RSG788 and RSG789)

Sequence 5’ tcg gtg gat acg gcg tgt tcg tcg tcg ttg gtg 3’
S V D T A C S S S L V

Mutation 5’ tcg gtg gat acg gcg **gct agc** tcg tcg ttg gtg 3’
S V D T A **A** S S S L V

2. AT mutant at S2672A (Primers: RSG790 and RSG791)

Sequence 5’ gtc atg ggc cac tcg atc ggt gag ttg tct gcg gca 3’
V M G H S I G E L S A A

Mutation 5’ gtc atg ggc cac **gcg** atc ggt gag **ctc** tct gcg gca 3’
V M G H **A** I G E L S A A

3. E20S mutant (Primers: RSG794 and RSG795)

Sequence 5’ aaa gcg ctg gtc cag gtg gaa cgc ctg aag cgc acc aac 3’
K A L V Q V E R L K R T N

Mutation 5’ aaa gcg ctg gtc cag **gtc tcg aga** ctg aag cgc acc aac 3’
K A L V Q V **S** R L K R T N

4. H7D mutant (Primers: RSG792 and RSG793)

Sequence 5’atg gtt gat caa ctc cag cat gcg acc gaa gcg ctg cgc 3’
M V D Q L Q H A T E A L R

Mutation 5’atg gtt gat caa **ctg** cag **gat** gcg acc gaa gcg ctg cgc 3’
M V D Q L Q **D** A T E A L R

5. E20S + H7D mutant (Primers: RSG792 to RSG795)

Sequence 5’atg gtt gat caa ctc cag cat gcg acc gaa gcg ctg cgc aaa gcg ctg gtc cag gtg gaa cgc ctg aag cgc acc3’
M V D Q L Q H A T E A L R K A L V Q V E R L K R T

Mutation 5’atg gtt gat caa **ctg** cag **gat** gcg acc gaa gcg ctg cgc aaa gcg ctg gtc cag **gtc tcg aga** ctg aag cgc acc 3’
M V D Q L Q **D** A T E A L R K A L V Q V **S** R L K R T
