# Supplementary material for: Novel Intermolecular Iterative Mechanism for Biosynthesis of Mycoketide Catalyzed by a Bimodular Polyketide Synthase
Source: PLoS Biol. 2008 Jul 8;6(7):e163. doi: 10.1371/journal.pbio.0060163 (PMC2443190; doi:10.1371/journal.pbio.0060163)
Supplement: Supplementary file 6 [file pbio.0060163.st002.pdf]

RSG 675	5’ tt cat atg gtt gat caa ctc cag cat gcg 3’
RSG 676	5’ tcc cac cgc cat gtg caa cgc cac 3’
RSG 677	5’ gtg gcg ttg cac atg gcg gtg gga 3’
RSG 678	5’ cga aag ccg ctg cag cac caa cat 3’
RSG 679	5’ tat ctg agc cag gcg cgc cac acc 3’
RSG 681	5’ aa aag ctt ctc gtc gtc gtt ccg aaa cgc 3’
RSG 788	5’ tcg gtg gat acg gcg gct agc tcg tcg ttg gtg 3’
RSG 789	5’ cac caa cga cga gct agc cgc cgt atc cac cga 3’
RSG 790	5’ gtc atg ggc cac gcg atc ggt gag ctc tct gcg gca 3’
RSG 791	5’ tgc cgc aga gag ctc acc gat cgc gtg gcc cat gac 3’
RSG 792	5’ atg gtt gat caa ctg cag gat gcg acc gaa gcg 3’
RSG 793	5’ cgc ttc ggt cgc atc ctg cag ttg atc aac cat 3’
RSG 794	5’ aaa gcg ctg gtc cag gtc tcg aga ctg aag cgc acc aac 3’
RSG 795	5’ gtt ggt gcg ctt cag tct cga gac ctg gac cag cgc ttt 3’
RSG1426	5’ tt gga tcc ctc ggc tca tca ccg caa gac act tca gcc3’
RSG1427	5’ aa cta ctg cag ctc gtc gtc gtt ccg aaa 3’
RSG1428	5’ tt ctg cag atg gtt gat caa ctc cag cat gcg 3’
RSG1430	5’ aa aag ctt cta gct cga gcg ctc cag caa cgc acg gtt 3’
RSG 1505	5’ tt cat atg ctc ggc tca tca ccg caa gac act tca gcc 3’
RSG1506	5’ gg aag ctt cta ctc gtc gtc gtt ccg aaa 3’
RSG1718	5’ tt cat atg gaa gga ctg ccc gaa gac gag 3’
